# CD9 Controls Integrin α5β1-Mediated Cell Adhesion by Modulating Its Association With the Metalloproteinase ADAM17

**DOI:** 10.3389/fimmu.2018.02474

**Published:** 2018-11-05

**Authors:** Yesenia Machado-Pineda, Beatriz Cardeñes, Raquel Reyes, Soraya López-Martín, Víctor Toribio, Paula Sánchez-Organero, Henar Suarez, Joachim Grötzinger, Inken Lorenzen, María Yáñez-Mó, Carlos Cabañas

**Affiliations:** ^1^Departamento de Biología Celular e Inmunología, Centro de Biología Molecular Severo Ochoa (CSIC-UAM), Madrid, Spain; ^2^Departamento de Biología Molecular, Facultad de Ciencias, Universidad Autónoma de Madrid, Madrid, Spain; ^3^Institute of Biochemistry, Christian-Albrechts University, Kiel, Germany; ^4^Department of Structural Biology, Institute of Zoology, Kiel, Germany; ^5^Centro de Biología Molecular Severo Ochoa, Instituto de Investigación Sanitaria la Princesa (IIS-IP), Madrid, Spain; ^6^Departamento de Inmunología, Oftalmología y OTR, Facultad de Medicina, Universidad Complutense, Madrid, Spain

**Keywords:** CD9, ADAM17, α5β1, integrin, tetraspanin, cell adhesion, fibronectin, metalloproteinase

## Abstract

Integrin α5β1 is a crucial adhesion molecule that mediates the adherence of many cell types to the extracellular matrix through recognition of its classic ligand fibronectin as well as to other cells through binding to an alternative counter-receptor, the metalloproteinase ADAM17/TACE. Interactions between integrin α5β1 and ADAM17 may take place both in *trans* (between molecules expressed on different cells) or in *cis* (between molecules expressed on the same cell) configurations. It has been recently reported that the *cis* association between α5β1 and ADAM17 keeps both molecules inactive, whereas their dissociation results in activation of their adhesive and metalloproteinase activities. Here we show that the tetraspanin CD9 negatively regulates integrin α5β1-mediated cell adhesion by enhancing the *cis* interaction of this integrin with ADAM17 on the cell surface. Additionally we show that, similarly to CD9, the monoclonal antibody 2A10 directed to the disintegrin domain of ADAM17 specifically inhibits integrin α5β1-mediated cell adhesion to its ligands fibronectin and ADAM17.

## Introduction

Integrins constitute an important family of heterodimeric (αβ) cellular receptors which, upon recognition and binding to specific ligands, mediate the adhesion of cells to components of the extracellular matrix (such as fibronectin, laminin, collagens) as well as cell-cell adhesion phenomena with crucial relevance in a variety of physiological and pathophysiological processes [reviewed in (1–3)]. Integrin α5β1 (also termed CD49e/CD29 or VLA5) binds to its canonical ligand fibronectin (Fn) through recognition of the Arg-Gly-Asp (RGD) motif in Fn-type-III module 10 and of a synergy site in Fn-type-III module 9, contributing to fibronectin assembly into fibrils (4). In addition to mediating cell adhesion through binding to its canonical Fn ligand, integrin α5β1 also has been reported to specifically recognize and bind to an alternative ligand, the disintegrin domain of ADAM17 (5–7).

ADAMs (A Disintegrin And Metalloproteinase) are a family of type-I transmembrane proteins with a modular structure that comprises the following domains (from N- to C-termini): a pro-, a catalytic-, a disintegrin-, a cysteine-rich-, and an EGF-like-domain, followed by a transmembrane- and a cytoplasmic region. 40 ADAMs have been identified in the mammalian genome from various species with the human genome containing 21 functional ADAMs, of which only 13 are proteolytically active while the rest lack the Zn-binding motif in the catalytic domain which is required for the proteolytic activity [reviewed in (8–10)]. Two closely related members of this family, ADAM10 and ADAM17, stand out among the catalytically active ADAMs as they are the two main cellular enzymes responsible for the cleavage and release of ectodomains from many cell surface proteins, a process known as “shedding” which plays an essential role in the development of tissues and organisms and in many other physiological as well as pathophysiological processes (11). ADAM10 and ADAM17 are also considered atypical members of the ADAM family since the extracellular cysteine-rich and EGF-like domains found in the rest of ADAMs are replaced in these two enzymes by a unique membrane proximal domain (MPD), which is involved in substrate recognition and binding as well as in regulation of their shedding activity [reviewed in (12, 13)].

All ADAMs contain a disintegrin domain in their extracellular region, which is structurally related to snake venom disintegrins. The disintegrin domains of ADAMs can potentially act as ligands for integrin binding, thus influencing cell adhesion and cell-cell interactions, with some degree of selectivity existing for these interactions between specific members of integrin and ADAM families (12–14).

Interactions of α5β1 with ADAM17 may occur among molecules expressed on the same cell (*cis*) or on different cells (*trans*), with the latter reported to support cell-cell adhesion events (6, 13). Interestingly, the interaction between integrin α5β1 and the disintegrin domain of ADAM17 has been shown to cause the inhibition of both the adhesive capacity of the integrin (i.e., its ability to bind its ligands) as well as that of ADAM17 metalloproteinase activity due to steric hindrance leading to decreased accessibility of its catalytic site for the substrates (6, 13). In contrast, stimuli that promote the dissociation of the α5β1-ADAM17 complex, such as an excess of soluble ADAM17 disintegrin domain, induce the activation of ADAM17 sheddase activity and enhance integrin adhesive capacity (6, 13).

The tetraspanin CD9, within the context of tetraspanin-enriched microdomains (TEMs), has been reported to associate on the cell surface with different adhesion receptors of the immunoglobulin and integrin families (15), including the integrin α5β1 (16–18). Through these interactions, CD9 exerts different regulatory effects on the function of associated adhesion molecules (19–23). On the other hand, CD9 also has been reported to associate directly with ADAM17 on the surface of different types of cells, thus exerting an inhibitory effect on ADAM17 sheddase activity against a variety of its substrates (19, 24–27).

Here we report that integrin α5β1 mediates the specific adhesion of different tumoral and leukocytic cells to immobilized recombinant ADAM17-Fc protein, which can be efficiently abrogated with blocking mAbs directed against the α5 or the β1 subunits of the integrin. Interestingly, the expression of CD9 on the cell surface or preincubation with mAb 2A10, which is directed to the disintegrin domain of human ADAM17, also abrogated the α5β1-mediated adhesion both to its canonical ligand Fn and to ADAM17-Fc. *In situ* proximity ligation assays (PLA) and biochemical experiments based on co-immunoprecipitation collectively revealed that the mechanism by which CD9 and mAb 2A10 inhibit α5β1-mediated cell adhesion is related to the reinforcement of *cis* interactions between ADAM17 and α5β1 on the cell surface, which takes place without alteration in α5β1 integrin affinity but is rather evidenced by changes in the organization of integrin molecules at the plasma membrane.

## Materials and methods

### Generation of mAB 2A10 against the disintegrin domain of human ADAM17

The mAb 2A10 was generated after mice immunization with the recombinant chimeric protein ADAM17-Fc, which encompasses the whole extracellular region of human ADAM17 fused to the Fc fragment of human IgG_1_, by employing the standard murine hybridoma technology. The experimental protocol followed was in accordance with the National Institutes of Health Guide for Care and Use of Laboratory Animals and was approved by the Animal Ethics Committee of the “Centro de Biología Molecular Severo Ochoa” (Madrid, Spain). The 2A10 mAb was selected from among the several hundred hybridomas generated based on its high and specific reactivity against ADAM17-Fc in ELISA assays. Assessment of the reactivity of 2A10 mAb against purified disintegrin (Dis) and membrane-proximal (MP) domains of human ADAM17, revealed that the epitope recognized by this mAb maps to the disintegrin domain.

### Cells and antibodies

Raji (Burkitt's lymphoma-derived B lymphoblastoid), JY (EBV-immortalized B lymphoblastoid), K562 (erythroblastic cell line), HSB2 (T lymphoblastic), Jurkat (T lymphoblastic), and Colo320 (colorectal adenocarcinoma) human cell lines were cultured in RPMI-1640. SKOV-3 (ovarian carcinoma) human cell line was grown in DMEM. LoVo (colorectal adenocarcinoma) human cell line was cultured in DMEM supplemented with F-12 nutrient mixture. All culture media were supplemented with 10% heat-inactivated FBS, 2 mM glutamine, 50 μg/ml streptomycin and 50 U/ml penicillin.

2A10 (anti-ADAM17); P1D6 (anti-α5 integrin) (28); TS2/16 (anti-β1 integrin), Lia1/2 (anti-β1 integrin) (29, 30), and HUTS21 (anti-β1 integrin) (31); TS1/18 (anti-β2 integrin) (32); PAINS-10 (anti-CD9) (33) and MEM-111 (anti-ICAM1/CD54) (34) mAbs were purified by protein A- or protein G-affinity chromatography. The A300D (specific for the disintegrin domain of human ADAM17) and A300E (specific for the membrane proximal domain of human ADAM17) mAbs have been described previously (35). When necessary, purified mAbs were biotinylated as previously described (33).

### Expression DNA constructs and CRISPR/Cas9-mediated gen knock out

For stable transfection experiments, Colo320 and HSB2 cells were incubated in 2.5% FCS–RPMI-1640 with the cDNA (20 μg) coding for human CD9 (in the pcDNA3 expression vector). Colo320 cells were electroporated at 412 V/cm and HSB2 cells at 200 V/cm (2 × 10 ms pulses in a 0.4 cm electroporation cuvette) in the ElectroSquarePorator ECM830 (BTX, Holliston,MA), positive clones were selected with G418 (0.8 mg/ml) in the culture medium (20).

To generate “Colo320-CRISPR ADAM17” and “Jurkat-CRISPR CD9” cell lines, cells were transfected with the CRISPR/Cas9 knockout plasmid pX461 encoding GFP and Cas9 nickase and the following sequences to generate the specific single guide RNAs: 5′-CACCGATCTAATATCCAGCAGCATT-3′ and 5′-CACCGTTTTTCTTACCGAATGCTGC-3′ for ADAM17 and 5′-CACCGTTCTTGCTCGAAGATGCTCT-3′ and 5′-CACCGGAATCGGAGCCATAGTCCAA-3′ for CD9. Transfected cells were sorted by flow cytometry based on their GFP transient fluorescence and then expanded and checked for suppression of ADAM17 or CD9 expression.

### Flow cytometry analysis

For flow cytometry analysis of protein surface expression cells were washed three times in RPMI-1640, incubated with primary antibodies at 4°C for 30 min followed by Alexa Fluor®647-conjugated anti-mousse IgG and fixed in 2% formaldehyde in PBS. Changes in integrin affinity were probed with the anti-β_1_ integrin activation reporter HUTS21 mAb. Cells were washed in cation-free medium (Hepes 20 mM, NaCl 149 mM, 2 mg/ml glucose) and incubated for 20 min at 37°C with Mn^2+^ (400 μM) or with Ca^2+^/Mg^2+^ (0.5 mM/1 mM, respectively) in the presence of biotinylated HUTS21 mAb. Cells were then washed and stained with Alexa Fluor®488-conjugated streptavidin. Fluorescence was measured using a FACScan™ flow cytometer (Beckton-Dickinson).

### Immunofluorescence, proximity ligation assays and confocal microscopy

For double immunofluorescence studies, cells were seeded on 12-mm diameter glass coverslips coated with poly-L-lysine (100 μg/ml). Cells were fixed in 2% formaldehyde (8 min at room temperature), blocked in 1% BSA in TBS (30 min at room temperature), and incubated for 1h with mAb 2A10 (10 μg/ml), followed by washes and incubation with the secondary antibody Alexa Fluor™-647-conjugated anti-mousse IgG (Thermo-Fisher Scientific). After incubation for 1 h with mouse serum (1/100) to block any free Fab binding sites of the secondary antibody, samples were incubated for 1 h with biotinylated-TS2/16 mAb (10 μg/ml), then with the secondary reagent streptavidin-Alexa Fluor™-488 (Life Technologies) and finally mounted on microscope slides with Fluoromount™/DAPI (Sigma-Aldrich).

*In situ* proximity ligation assays (PLA; Duolink kit, Sigma Aldrich) allows detection of direct protein–protein interactions in cell samples by fluorescence microscopy (36). Colo320 cells were seeded, fixed and blocked as described above. Next, samples were incubated simultaneously with 2A10 (anti-ADAM17) mouse mAb and with an anti-α5 rabbit polyclonal antibody, followed by specific oligonucleotide-labeled secondary antibodies (anti-mouse plus and anti-rabbit minus probes). Only if the two target proteins are in close proximity (≤40 nm), the oligonucleotides of the two probes will hybridize and after a rolling-circle amplification reaction and detection with a different fluorescently labeled oligonucleotide, fluorescent dot signals can be visualized by microscopy. Samples were mounted with ProLong® anti-fade reagent and images were obtained with a Leica LSM510 inverted confocal microscope. Fiji/Image-J software was used for detection and analysis of fluorescent dots.

### Co-immunoprecipitation

Co-immunoprecipitation experiments were performed using intact cells, in order to detect only surface protein–protein interactions. Cells were incubated for 1 h at RT with the anti-β1 mAb Lia1/2 or the anti-CD9 mAb PAINS-10 in the presence of Ca^2+^+Mg^2+^ (500 μM + 500 μM) or Mn^2+^ (200 μM), followed by washing the non-bound antibody excess. Cells were then lysed for 15 min at 4°C in TBS containing 1% Brij-97 in the presence of corresponding extracellular cations and protease inhibitors and, after removal of insoluble material, incubated for 4 h at 4 °C with protein A-sepharose. Beads were then washed with 1:5 diluted lysis buffer, boiled in nonreducing (for detection of CD9 and β1 integrin) or reducing (for detection of ADAM17) Laemmli buffer, resolved by 8% or 12% SDS-PAGE and transferred onto nitrocellulose membranes. Membranes were blocked with 3% BSA and developed either with biotinylated anti-CD9 mAb PAINS10 or biotinylated anti-β1 mAb TS2/16 or anti-ADAM17 mAb A300D, followed by incubation respectively with streptavidin-HRP (Thermo-Fisher Scientific) or anti-mouse IgG-HRP (Sigma-Aldrich) secondary reagents and ECL-chemiluminescence detection on an ImageQuant LAS4000-mini system.

### Cell adhesion assays

**Figure 1:**
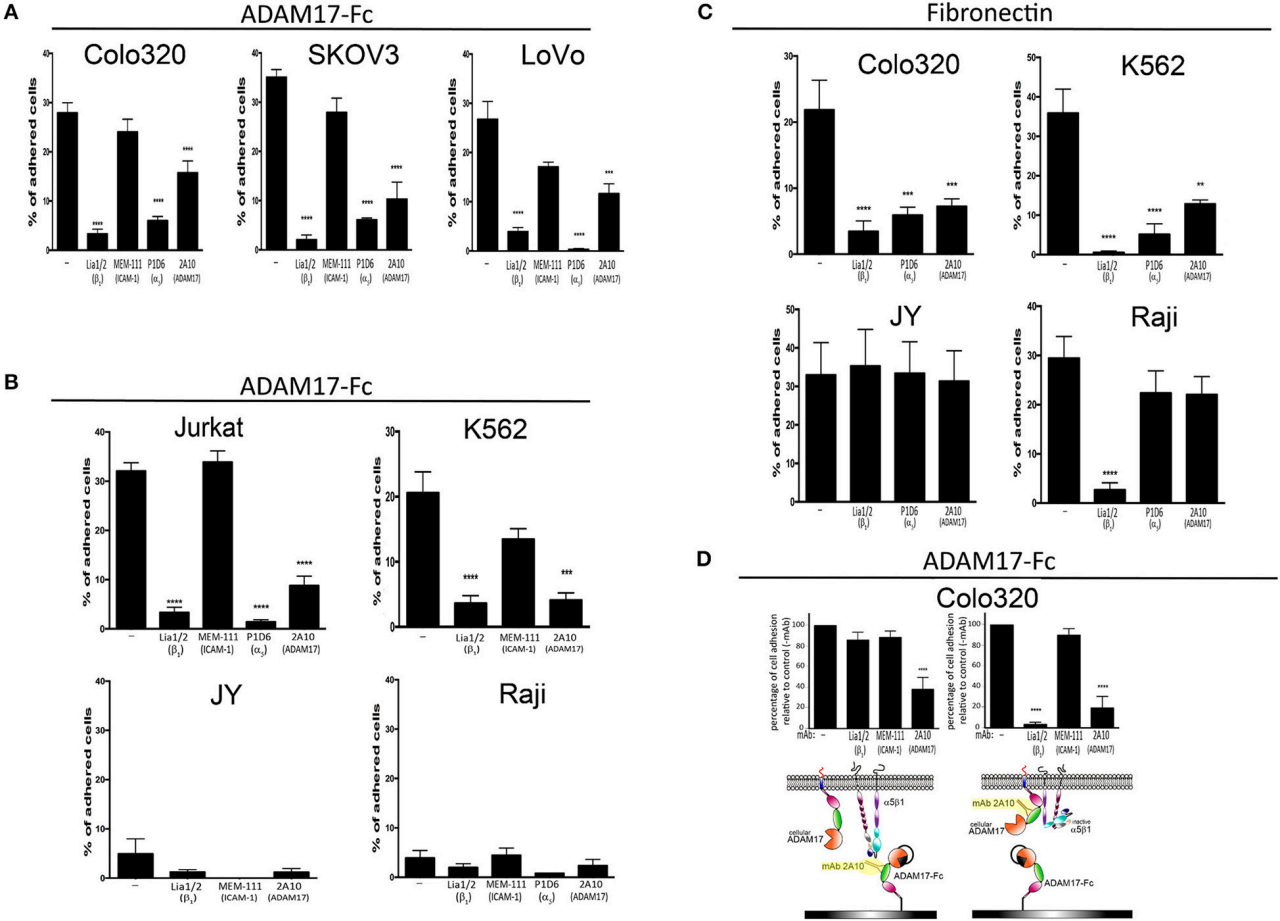
The adhesion of different tumoral and leukocytic cells to Fn and ADAM17-Fc is specifically mediated by integrin α5β1. The adhesion of different human cell lines derived from solid tumors **(A)** or hematopoietic malignancies **(B)** to immobilized ADAM17-Fc or to fibronectin **(C)** is represented. The effect of mAbs Lia1/2 (blocking anti-β1 integrin subunit), P1D6 (blocking anti-α5 integrin subunit) and mAb 2A10 (specific for the disintegrin domain of human ADAM17) on cell adhesion is shown. The anti-ICAM1/CD54 mAb MEM 111 was included as a negative control. Data show the percentages of adherent cells (means ± SEM of at least three different experiments, each performed in triplicates). **(D)** Effect of preincubating plastic-immobilized ADAM17-Fc (left graph) or Colo320 cells (right panel) with the indicated mAbs (the excess non-bound antibody was subsequently washed away) prior to adding the cells to the ADAM-17-coated wells and allowing their adhesion at 37°C. In all cases, cells were stimulated with PMA (200 ng/ml) for 2 h, loaded with the fluorescent probe BCECF-AM and then allowed to adhere to plastic-immobilized ligands ADAM17-Fc (20 μg/ml) or Fn (7.5 μg/ml) for 60 min at 37°C in the presence of Mn^2+^ (200 μM). Data show the percentages of adherent cells (means ± SEM of three experiments, performed in triplicates) relative to the 100% cell adhesion considered in the absence of antibody treatment. Data were analyzed by one-way ANOVA with Dunnett's post-test analysis **p < 0.01, ***p < 0.001 and ****p < 0.0001 values denote the statistical significance of differences between a specific condition and the control condition in the absence of antibody (−).

Static cell adhesion to ADAM17-Fc, ICAM1-Fc or fibronectin-coated wells was performed as described previously (19, 37). 96-well flat-bottom plates were pre-coated overnight at 4°C with ADAM17-Fc (20 μg/ml), ICAM1-Fc (20 μg/ml) or fibronectin (7.5 μg/ml) and blocked for 2 h with 1% BSA in PBS. For PMA-stimulated cell adhesion, cells were incubated with PMA (200 ng/ml) in RMPI-1640 for 2h at 37°C. Cells (2 × 10^5^ cells/well) were loaded with the fluorescent probe BCECF-AM for 20 min at 37°C in PBS, washed, resupended in adhesion buffer (Hepes 20 mM, NaCl 149 mM, 2 mg/ml glucose) containing 200 μM Mn^2+^, added to the wells in the presence of the appropriate mAbs (10 μg/ml) and allowed to adhere for 60 min at 37°C. When indicated in Figure 1D, only the cells or the plates precoated with immobilized ligands ADAM17-Fc or Fn were incubated first with 10 μg/ml of anti-ADAM17 (2A10), anti-β_1_ (Lia1/2), anti-α_5_ (PID6), or the control anti-ICAM1 (MEM-111) mAbs for 60 min at 4°C, then the excess non-bound antibody was washed and the cells were subsequently allowed to adhere onto the immobilized ligand by transferring the plates to 37°C for 60 min. After gently washing the wells several times with PBS at 37°C to remove non-adherent cells, the percentage of adherent cells in each well was calculated by determining their fluorescence in a microplate reader (TecanGENios) before and after having removed the non-adherent cells.

### Statistical analysis

As indicated in the individual figure legends, different statistical analyses of data were performed depending on the quantitative and qualitative nature of the variables being considered. These analyses include the two-tailed paired *T*-test and the one-way ANOVA coupled with Dunnet's, Tukey's, or Šidák's multiple comparison tests.

## Results

### Integrin α5β1 mediates the adhesion of tumoral and leukocytic cells to immobilized ADAM17

Recombinant ADAM17 has been reported to support integrin α5β1-dependent fibroblast and kidney mesangial cell adhesion, and such adhesion was demonstrated to occur through integrin binding to the disintegrin domain of ADAM17 (5–7). We decided to build on these findings by assessing whether the adhesion of several other human cell lines derived either from solid tumors (“cancer cell lines”) or hematological malignancies (“leukocytic cell lines”) to immobilized recombinant ADAM17-Fc was also mediated by integrin α5β1. Figure 1A shows that, when stimulated with phorbol ester PMA and divalent cation Mn^2+^ (a potent activating agent that induces integrin high affinity state), Colo320 (colorectal adenocarcinoma), LoVo (colorectal adenocarcinoma) and SKOV-3 (ovarian carcinoma) cancer cells readily and specifically adhered to a substrate coated with ADAM17-Fc. In all cases, adhesion of these cells to ADAM17-Fc was specifically mediated by integrin α5β1, as demonstrated by the potent inhibition achieved either with a blocking anti-α5 mAb (P1D6) or anti-β1 mAb (Lia1/2) antibodies. Likewise, the PMA/Mn^2+^-stimulated adhesion of leukocytic Jurkat (T lymphoblastic) and K562 (erythroblastic) cells to ADAM17-Fc was also shown to be fundamentally dependent on integrin α5β1 (Figure 1B). In contrast, the leukocytic JY (EBV-immortalized B lymphoblastoid cell line) cells, which do not express any β1-containing integrins (including α5β1) due to the lack of expression of this integrin chain, and Raji (Burkitt's lymphoma-derived B lymphoblastoid cell line) cells, which do not express integrin α5β1 although they express abundant α4β1 (another important Fn receptor), only displayed a negligible level of adhesion to ADAM17-Fc (<5%), even after strong stimulation with PMA/Mn^2+^.

Colo320 and K562 cells, which selectively express abundant levels of the integrin α5β1 on their surface, also adhered efficiently to Fn after stimulation with Mn^2+^ and this adhesion was blocked by either P1D6 or Lia1/2 mAbs. However, although JY and Raji cells also adhered very efficiently to Fn, such adhesion was not inhibited by the blocking anti-α5 (P1D6) mAb as it was not mediated by integrin α5β1, but rather through integrins α4β7 and α4β1, respectively (Figure 1C).

Taken together, these results concur with previous reports (5–7) and confirm that integrin α5β1, in addition to binding to its canonical ligand Fn, also mediates the adhesion of a wide variety of cells to immobilized ADAM17-Fc.

**Figure 2:**
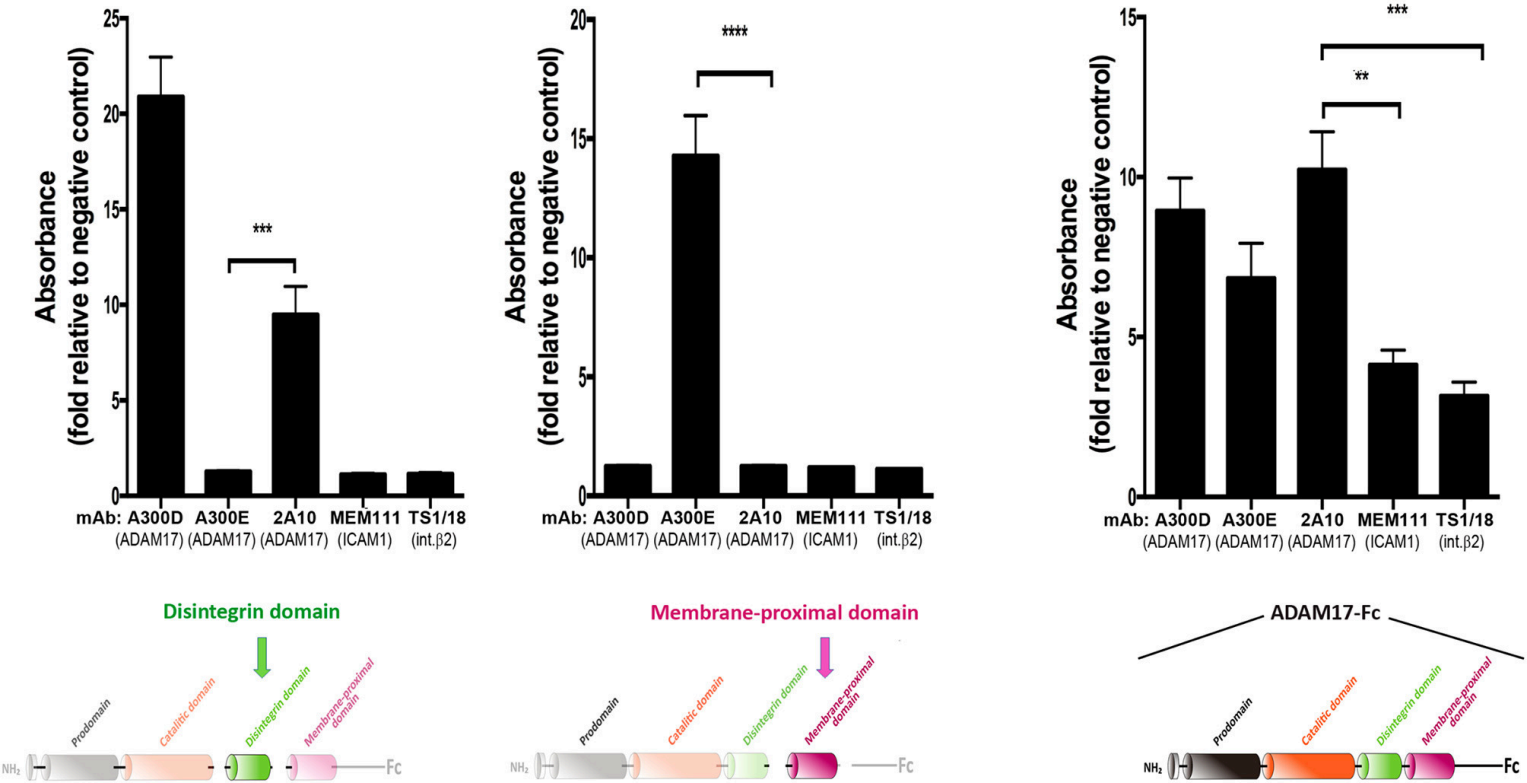
Monoclonal antibody 2A10 is specific for the disintegrin domain of human ADAM17 (hADAM17). Assessment by ELISA of the reactivity of mAb 2A10 against the purified disintegrin domain of hADAM17 **(left)**, the membrane-proximal domain of hADAM17 **(middle)** or the recombinant chimeric protein ADAM17-Fc **(right)**. The mAb A300D, which recognizes an epitope mapped to the disintegrin domain of human ADAM17 and the mAb A300E, which recognizes an epitope mapped to the membrane-proximal domain of human ADAM17, were used respectively as positive controls for each of these domains. The mAb TS1/18 (anti-human β_2_ integrin) and mAb MEM111 (anti-ICAM1/CD54) were used as negative controls. Graphs show the absorbance, expressed in arbitrary units, relative to the unspecific absorbance background in the absence of antibody. Data correspond to the mean ± SEM from three independent experiments, each performed in triplicate and analyzed by one-way ANOVA with Dunnett's post-test analysis **p < 0.01, ***p < 0.001, ****p < 0.0001. The colored schematics underneath each panel highlight the specific domains of hADAM17 immobilized on plastic for each ELISA assay.

The mAb 2A10 was generated in our laboratory and shown by ELISA assays to be specific against an epitope located on the disintegrin domain of human ADAM17 mAb (Figure 2).

We then assessed the effects of mAb 2A10 on cell adhesion to both α5β1 ligands, ADAM17-Fc and Fn. As shown in Figure 1, in all cases where cell adhesion was predominantly mediated by integrin α5β1, mAb 2A10 exerted a potent inhibitory effect on cell adhesion. In contrast, mAb 2A10 had no effect on the adhesion of JY or Raji cells to Fn which, as indicated above, is not mediated by α5β1. Furthermore, mAb 2A10 had no effect on the cell adhesion mediated by integrin LFA-1 (αLβ2), clearly showing that its effects are specifically mediated through integrin α5β1 (Supplementary Figure 1).

The observed blockade of cell adhesion to Fn with mAb 2A10 suggested that this antibody must exert its effects on α5β1-mediated adhesion through a *cis*-type mechanism after binding to cell surface ADAM17. In any case, and to rule out a possible steric hindrance of cell adhesion caused by binding of mAb 2A10 to the disintegrin domain of the immobilized ligand ADAM17-Fc, we first incubated Colo320 cells with 2A10 and washed away the excess of unbound antibody before allowing cells to adhere onto immobilized ADAM17-Fc (Figure 1D, right panel). These analyses confirmed that the adhesion blocking effect exerted by mAb 2A10 was due to a regulation in *cis* of α5β1-mediated adhesion.

### Expression of CD9 inhibits α5β1-mediated cell adhesion to ADAM17 and fibronectin

**Figure 3:**
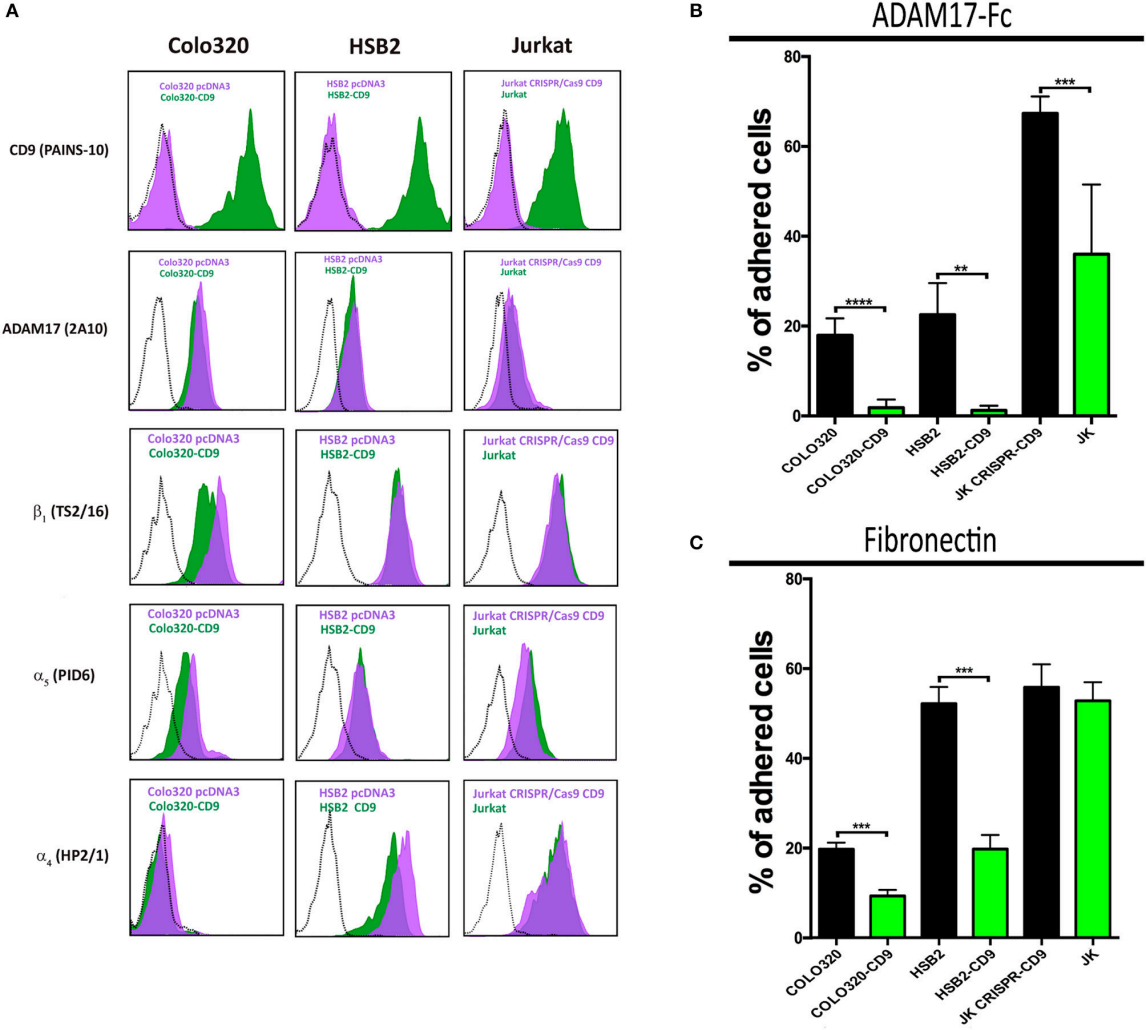
Ectopic expression or knocking-out of CD9 regulates α5β1-mediated cell adhesion to ADAM17-Fc and Fn. Flow cytometric detection of cell surface CD9 (mAb PAINS-10), ADAM17 (mAb 2A10), β_1_ (mAb TS2/16), α5 (mAb P1D6), and α4 (mAb HP2/1) on Colo320, HSB2 and Jurkat cells **(A)**. The black dotted line histograms correspond to negative controls, green filled histograms correspond to the expression of the indicated molecules on CD9 expressing cells (Colo320-CD9, HSB2-CD9, Jurkat) and purple filled histograms correspond to the expression of the indicated molecules on their respective CD9-lacking counterparts (Colo320, HSB2, Jurkat-CRISPR-CD9). The percentages of adhesion of paired variants of Colo320, HSB2 and Jurkat cells, either expressing CD9 (Colo320-CD9, HSB2-CD9, Jurkat; green bars) or lacking expression of this tetraspanin (Colo320, HSB2, Jurkat CRISPR-CD9; black bars) on plastic-immobilized ADAM17-Fc **(B)** or on fibronectin **(C)**. In all cases, cells were stimulated with PMA (200 ng/ml) for 2 h, loaded with the fluorescent probe BCECF-AM and then allowed to adhere to plastic-immobilized ligands ADAM17-Fc (20 μg/ml) or Fn (7.5 μg/ml) for 60 min at 37°C under conditions of integrin activation in the presence of Mn^2+^ (200 μM). Data are presented as the percentage of adhered cells (means ± SEM of three experiments each performed in triplicates). Statistical analysis was carried out using the two-tailed paired *T*-test. **, *** and **** denote *p* < 0.01, *p* < 0.001 and *p* < 0.0001, respectively.

We have previously shown that CD9 on the cell surface is engaged in direct interactions with ADAM17 and through such association inhibits ADAM17 metalloproteinase activity (19, 24, 25). Therefore, we decided to assess whether the presence or absence of CD9 on the cell surface could influence not only ADAM17 sheddase activity but also its regulatory effect exerted on the adhesive activity of integrin α5β1. For this purpose, we generated a series of paired variants derived from Jurkat, HSB2 and Colo320 cell lines, that differed in the presence/absence of CD9 following either the neoexpression of this tetraspanin (by stable transfection of CD9 cDNA in CD9^−^ Colo-320 and HSB2 cells) or its supression by CRISPR/Cas9 gene knockout (in CD9^+^ Jurkat cells; Figure 3A). For all these three cell lines, the variants expressing CD9 (Colo320-CD9, HSB2-CD9, Jurkat) displayed a greatly reduced capacity to adhere to ADAM17-Fc, even in the presence of the potent integrin stimulus Mn^2+^, compared to their respective CD9^−^ (Colo320, HSB2, Jurkat-CRISPR-CD9) counterparts (Figure 3B). We also assessed the adhesion of these paired cell variants to Fn and again, the presence of CD9 resulted in reduced cell adhesion (Figure 3C), except for the case of Jurkat cells that, in addition to α5β1, also express very high levels of the fibronectin-binding integrin α4β1 (Figure 3A).

### CD9-induced inhibition of α5β1-mediated cell adhesion cannot be attributed to decreased integrin conformational change to the high affinity state

**Figure 4:**
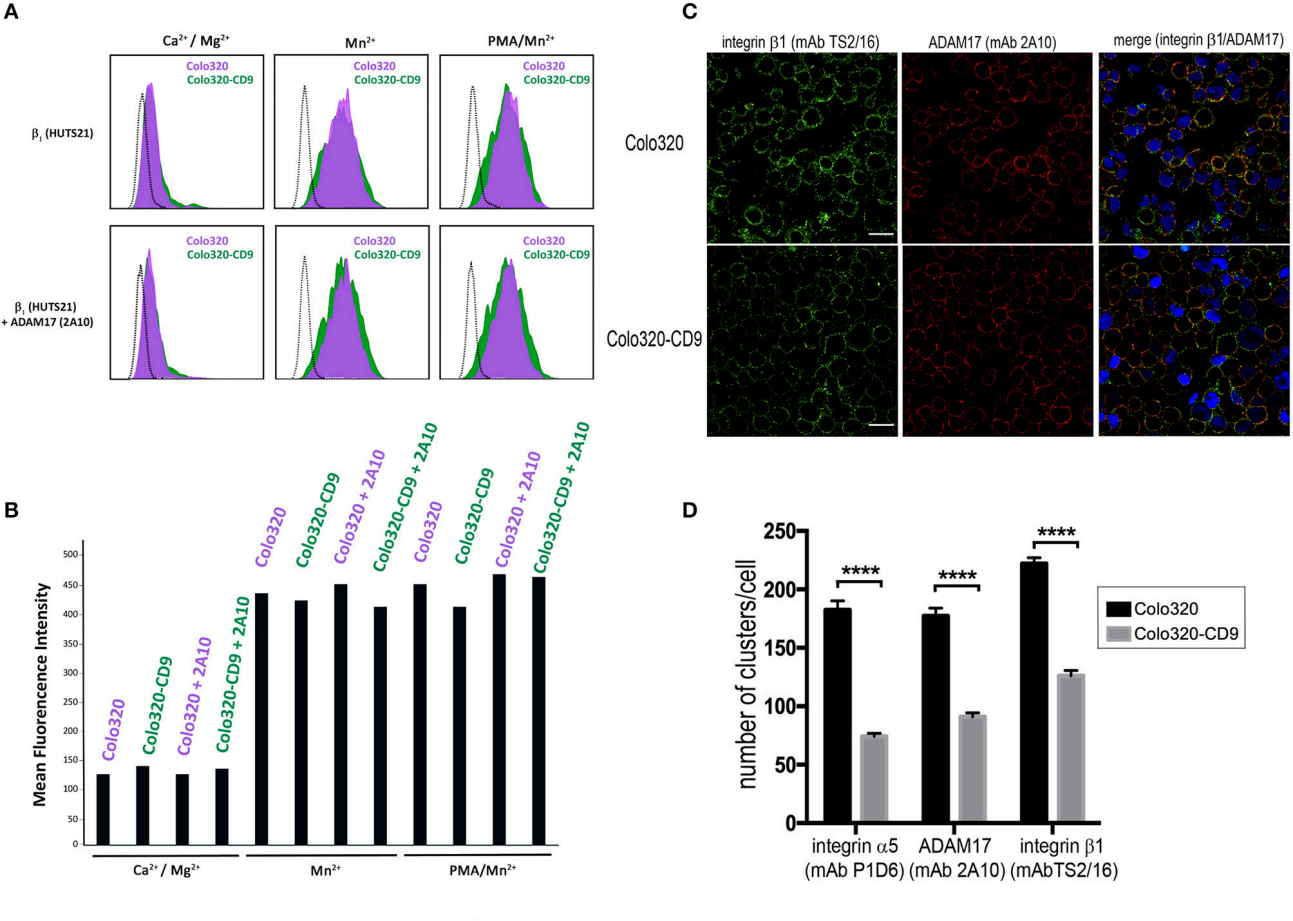
CD9-induced inhibition of integrin α5β1-mediated cell adhesion does not involve alterations of integrin affinity. **(A)** Flow cytometric analysis of the expression of epitope HUTS21 in different integrin activation conditions: Ca^2+^/Mg^2+^ (0.5 mM/1mM, respectively), Mn^2+^ (200 μM) or PMA/Mn^2+^ (200 ng/ml/200 μM, respectively) in Colo320 (purple-filled histograms) and Colo320-CD9 (green-filled histograms). The lower row of histograms represents the effect of cell pre-incubation with the mAb 2A10 on the expression of HUTS21 epitope in Colo320 and Colo320-CD9 cells. The black dotted line histograms correspond to negative controls. The fluorescence of 2,000 cells was acquired in the flow cytometer for each histogram. **(B)** Expression of HUTS21 epitope represented as the mean florescence intensities from the flow cytometry histograms shown in **(A)**. **(C)** Double immunofluorescence staining and confocal microscopy analysis showing the effects of CD9 expression on the organization of integrin α5β1 (green) and ADAM17 (red) molecules on the surface of Colo320 cells. Colo320 and Colo320-CD9 cells were stained with the anti-β1 (TS2/16) or anti-ADAM17 (2A10) mAbs. Representative images of confocal sections are shown. Scale bar = 20 μm. **(D)** Quantitation of the number of clusters/cell of integrin α5, β1, and ADAM17 molecules on the surface of Colo320 and Colo320-CD9. Graph shows the means ± SEM of the number of clusters/cell, calculated from at least 350 different cells for each condition using the Image-J thresholding and particle analyses.****denotes *p* < 0.0001 in a one-way ANOVA with Tukey's post-test.

The observed abrogation of integrin α5β1-mediated cell adhesion caused by the cell surface expression of CD9 could be due either to diminished integrin affinity or to an alteration in the organization of α5β1 integrin molecules on the cell surface resulting in lower multivalent avidity for ligand. First we investigated whether changes in the affinity of α5β1 were being induced by the presence of CD9, and for this purpose we compared in Colo320 and Colo320-CD9 cells the expression of the epitope HUTS21, which reports the high affinity state—characterized by an extended and open headpiece conformation—of integrins that contain the β1 subunit (31), and particularly of integrin α5β1 (38, 39). Colo320/Colo320-CD9 cells represent a very clean cellular system to assess α5β1 integrin-mediated adhesion, since they selectively express high levels of α5β integrin, but neither α4β1 integrin nor any other β2-associated integrins, and only very low levels -if at all- of other major integrins (not shown). As shown in Figures 4A,B, no significant differences in the expression of the HUTS21 epitope between Colo320 and Colo320-CD9 cells were detected, neither in the presence of extracellular Ca^2+^ and Mg^2+^, which resemble resting physiological conditions in terms of integrin activation, or under conditions which fully induce the acquisition of integrin high affinity such as the presence of Mn^2+^ or the combination of Mn^2+^ and phorbol ester PMA. Moreover, pretreatment with mAb 2A10 did not change the expression of HUTS21 epitope relative to cells not preincubated with 2A10 under any of the conditions tested (Ca^2+^/Mg^2+^, Mn^2+^, or Mn^2+^/PMA). Taken together, these results indicate that the inhibitory effects caused by the presence of CD9 or by preincubation with mAb 2A10 on the adhesive activity of integrin α5β1 are not primarily mediated by alterations in the ability of integrin molecules to switch to the high affinity conformation.

We also investigated whether the observed inhibitory effects on α5β1-mediated cell adhesion induced by the presence of CD9 are caused by changes in the organization of α5β1 molecules on the cell surface. Double immunofluorescence staining of integrin α5β1 and ADAM17 molecules in CD9-negative (Colo320) or CD9-positive (Colo320-CD9) cells using mAbs specific for integrin α5β1 (anti-β1 subunit mAb TS2/16) or ADAM17 (mAb 2A10) and analysis by confocal microscopy revealed a certain degree of colocalization between α5β1 and ADAM17 in both Colo320 and Colo320-CD9 cells, with Pearson's coefficients over 0.6 (not shown). However, a different pattern of integrin and ADAM17 distribution was observed depending on whether cells expressed or not CD9 on the cell surface (Figure 4C), displaying a dotty distribution in Colo320 cells and a more homogeneous appearance on the surface of Colo320-CD9 cells. Quantitation of the number of clusters of α5, β1 and ADAM17 molecules revealed that on cells expressing CD9 (Colo320-CD9) the number of clusters was significantly reduced, indicating that the presence of CD9 favors the formation of larger clusters containing both α5β1 and ADAM17 molecules (Figure 4D).

**Figure 5:**
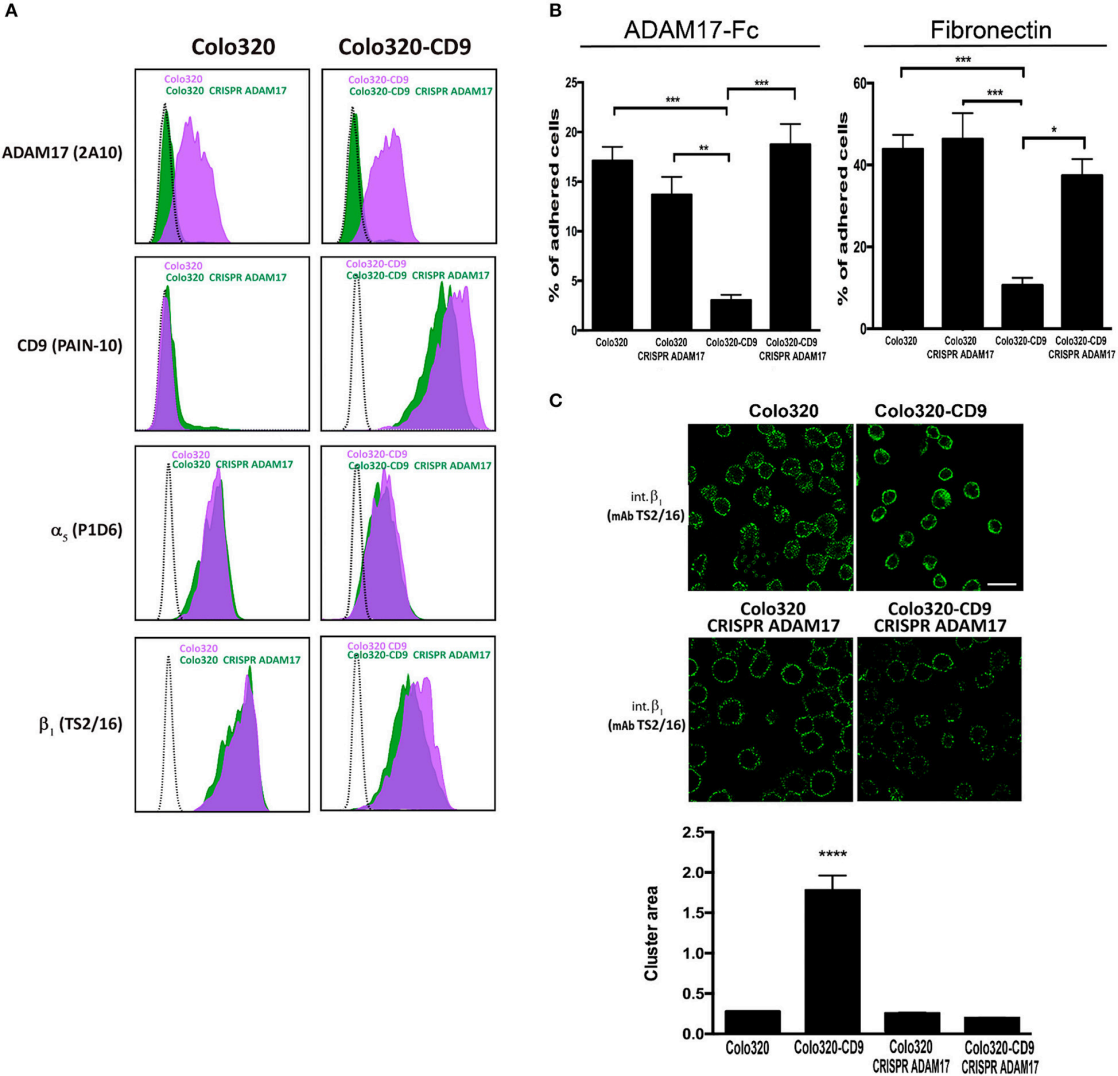
Knocking out ADAM17 reverts the CD9-mediated abrogation of α5β1-dependent cell adhesion and the alteration of α5β1 distribution on the cell surface. **(A)** Flow cytometric detection of ADAM17 (mAb 2A10), CD9 (mAb PAINS-10), α5 (mAb P1D6) and β1 (mAb TS2/16) on the surface of Colo320 and Colo320-CD9 cells either expressing or lacking ADAM17 expression. The dotted black line histograms correspond to negative controls, green-filled histograms show the expression of the indicated molecules on ADAM17-lacking cells (Colo320 CRISPR ADAM17 and Colo320-CD9 CRISPR ADAM17) and purple-filled histograms show to the expression of the indicated molecules on Colo320 and Colo320-CD9 (ADAM17-positive cells). For each histogram the fluorescence signal of 2000 cells was acquired in the flow cytometer. **(B)** Cell adhesion of ADAM17-positive and ADAM17-negative Colo320 and Colo320-CD9 cells. Cells were stimulated with PMA (200 ng/ml) for 2 h, loaded with the fluorescent probe BCECF-AM and then allowed to adhere to immobilized ligands ADAM17-Fc (20 μg/ml) or Fn (7.5 μg/ml) for 60 min at 37°C under conditions of integrin activation in the presence of Mn^2+^ (200 μM). The percentage of cells that remained adhered is indicated as mean ± SEM. Statistical analysis performed was one-way ANOVA and Tukey's multiple comparison test. *p < 0.05, **p < 0.01, ***p < 0.001. **(C)** Immunofluorescence staining and confocal microscopy analysis of α_5_β_1_ integrin molecules organization on the surface of ADAM17-positive and ADAM17-negative Colo320 and Colo320-CD9 cells. α5β1 molecules were stained with the anti-β1 (TS2/16) mAb. Representative images of confocal microscopy sections are shown. Scale bar = 20 μm. Lower panel: Quantitation of the area of integrin α_5_β_1_ clusters in square micrometers on the surface of the different types of cells. Graph depicts the means ± SEM of cluster areas calculated from 800-3000 different cells using the Image J software. ****denotes *p* < 0.0001 in a one-way ANOVA with Šidák's post-test.

The association between α5β1 and ADAM17 has been reported to result in inhibition of the activity of both molecules. *Vice versa*, the induction of the activation of either integrin α5β1 or ADAM17 causes their dissociation from the complex (6, 13). We therefore postulated that the inhibitory effect on α5β1-mediated cell adhesion exerted by the presence of CD9 could be due to a reinforcement of the *cis* interaction between α5β1 and ADAM17 caused by this tetraspanin. If this was the case, the inhibition of α5β1-mediated adhesion by CD9 expression, should not be observed in the absence of ADAM17. To directly assess this hypothesis, ADAM17 expression was knocked-out using the CRISPR/Cas9 technique both in Colo320 and Colo320-CD9 cells (Figure 5A). Interestingly, when ADAM17 was absent in Colo320-CD9 cells, these cells recovered their full capacity to adhere efficiently to both ADAM17-Fc and Fn through integrin α5β1 (Figure 5B). In addition, the distribution of α5β1 molecules was also restored showing again an organization in smaller and more dispersed clusters, similar in size to those on Colo320 cells (Figure 5C). Taken together, our results indicate that CD9 exerts an important control on both the distribution and the adhesive capacity of α5β1 integrin molecules.

**Figure 6:**
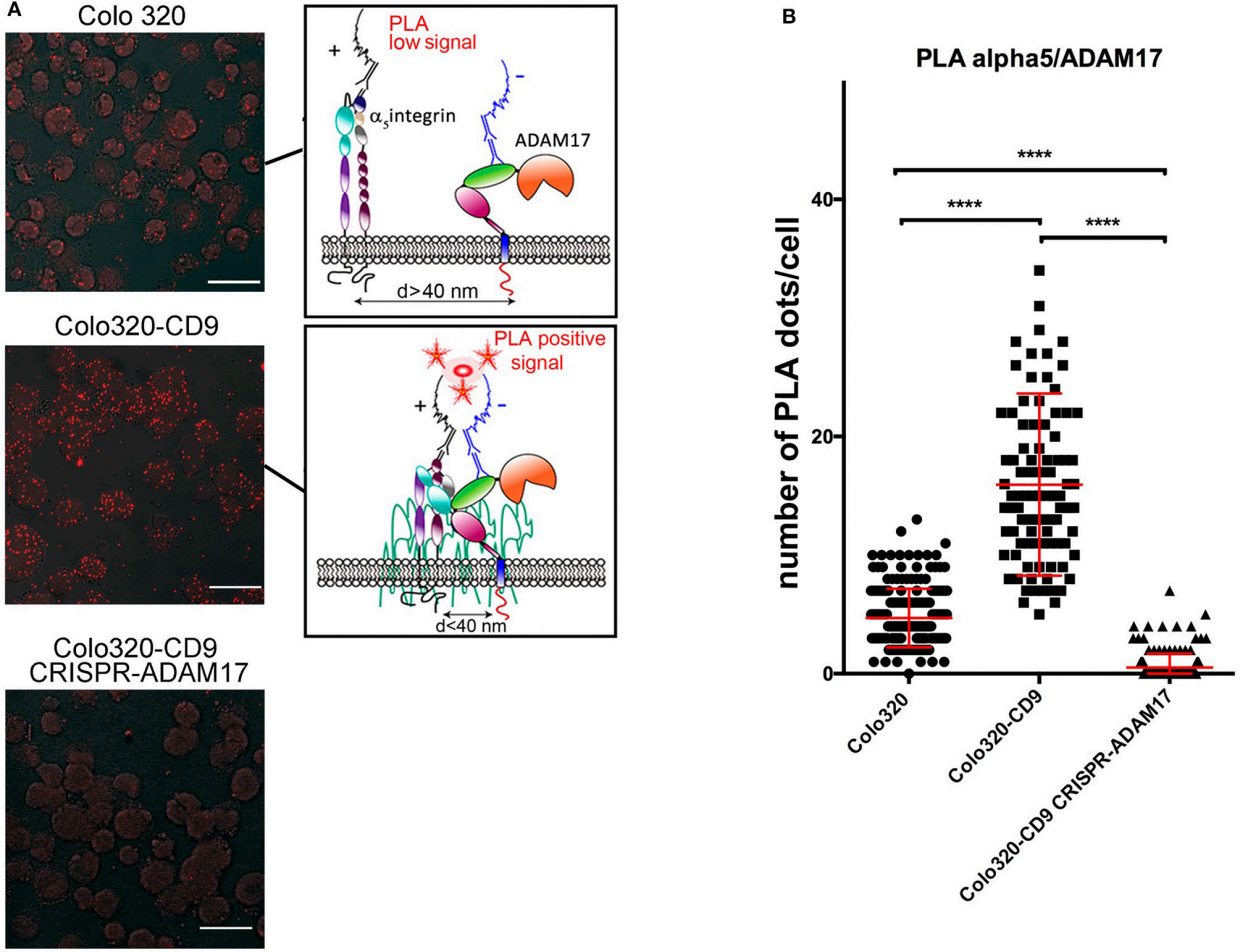
Effects of the expression of CD9 on the *cis* interactions between integrin α_5_β_1_ and ADAM17. **(A)** Analysis by *in situ* proximity ligation assays (PLA) of the molecular association of ADAM17 with integrin α5β1 on cells expressing CD9 (Colo320-CD9, middle image) or not (Colo320, top image). Cells expressing CD9 but lacking ADAM17 (Colo320-CD9/CRISPR-ADAM17, bottom image) were included as an internal negative control for ADAM17-α5 association. Scale bars = 20 μm. Sketches on the right summarize the principle of PLA. **(B)** The graph represents the mean ± SEM of the number of PLA dots/cell calculated from at least 100 cells (for each cell line) from different micrographic fields in two different experiments and analyzed by one-way ANOVA with Tukey's post-test analysis. ****p < 0.0001.

However, clustering analysis by confocal microscopy provide insight only about the high-order organization and subcellular localization of molecules in the cell. To explore the organization α5β1 integrin and ADAM17 at the plasma membrane closer to the molecular level, we chose to use *in situ* Proximity Ligation Assays (PLA), that have been previously employed to demonstrate the cis interaction between ADAM17 and integrin α5β1 (6). PLA provide positive signals (“PLA fluorescent dots”) only if the two proteins under analysis are in close proximity (typically <40 nm) that is compatible with a direct interaction between them. To quantitatively analyze the effect of CD9 expression on the α5β1/ADAM17 interactions, PLA were performed to detect the interactions between ADAM17 and α5β1 on both Colo320 and Colo320-CD9 cells. As shown in the images of Figure 6A, the number of PLA dots was clearly higher on Colo320-CD9 cells and a detailed quantitative analysis revealed a 5-fold increment in the number of dots/cell on CD9-positive cells (Colo320-CD9) compared to CD9-negative cells (Colo320; Figure 6B). As a relevant negative control for these experiments, when ADAM17 was knocked-out in Colo320-CD9 cells (Colo320-CD9 CRISPR-ADAM17), no PLA dots were observed. These results provide strongly support for the *cis* α5β1-ADAM17 association being enhanced on the cell surface by the presence of CD9, probably through the formation of ternary complexes among these molecules (α5β1:CD9:ADAM17).

**Figure 7:**
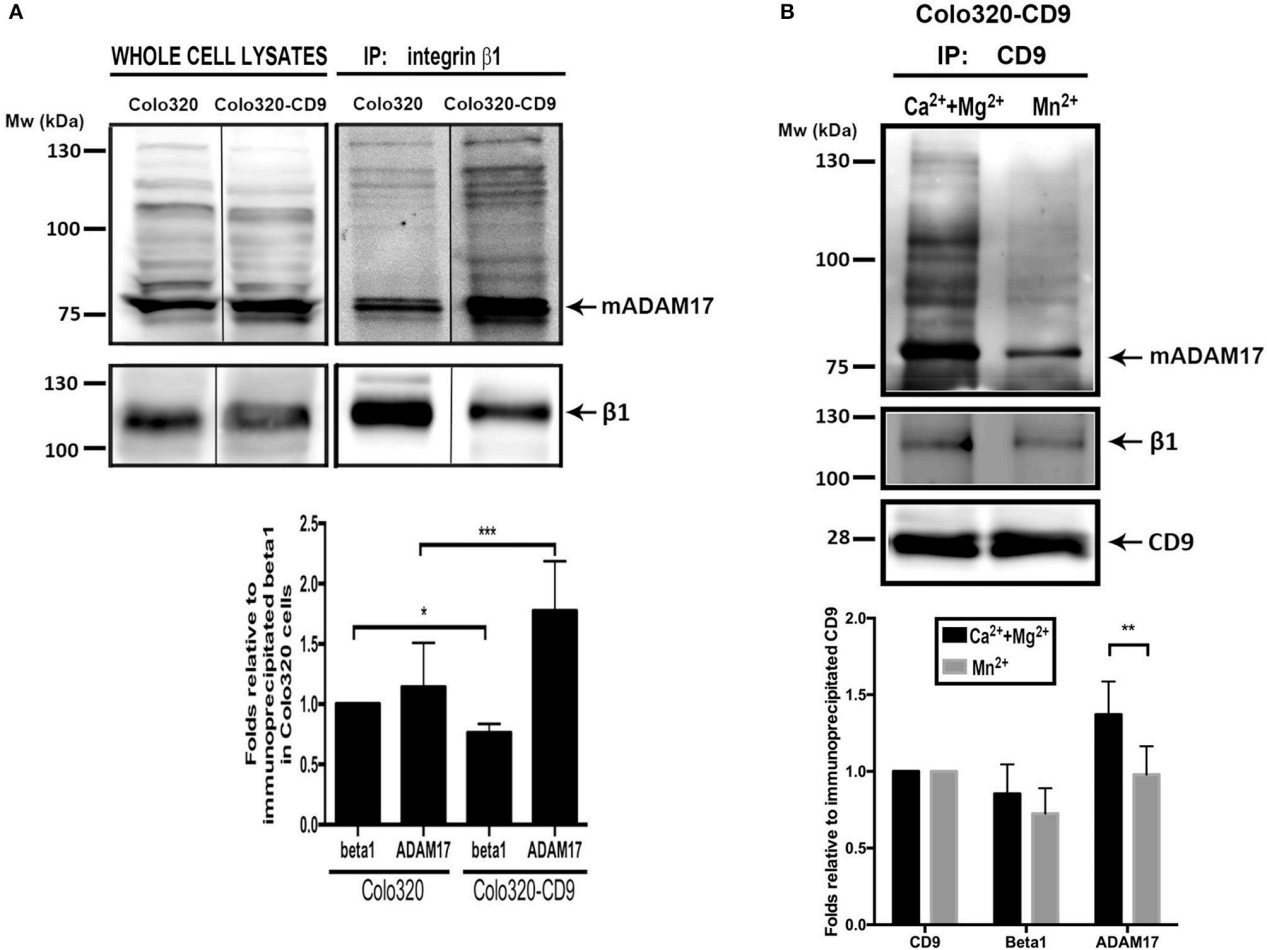
Cell surface ADAM-17 is more efficiently co-immunoprecipitated with integrin α5β1 from Colo320-CD9 cells than from Colo320 cells. **(A)** Only cell surface integrin α_5_β_1_ molecules were selectively immunoprecipitated by incubating the cells with mAb Lia1/2 (anti-β_1_) in the presence of Ca^2+^+Mg^2+^ (500 μM each) and washing the excess non-bound antibody prior to cell lysis and immunoprecipitation. Immunoprecipitated integrin α_5_β_1_ and co-immunoprecipitated ADAM17 were detected by immunoblotting with the anti-β1 (TS2/16) and anti ADAM17 (A300D) mAbs, respectively. The gel shown is representative of five different experiments. The graph below shows the densitometric quantitation of the amount of precipitated integrin and co-immunoprecipitated ADAM17 (means ± SEM) from five different experiments, normalized to β_1_ precipitated in Colo320 cells in each experiment. **(B)** Integrin α5β1 and ADAM17 are coimmunoprecipitated with CD9 from cell surface TEMs. CD9 was immunoprecipitated with mAb PAINS10 as described for integrin β1 in **(A)** but under two different extracellular cation conditions: in the presence of Ca^2+^+Mg^2+^ (500 μM each) or Mn^2+^ (200 μM). Immunoprecipitated CD9 and co-immunoprecipitated β_1_ and ADAM17 were detected by immunoblotting with mAbs PAINS10 (anti-CD9), TS2/16 (anti-β1), and A300D (anti ADAM17), respectively. The gel shown is representative of four different experiments. The graph below represents the densitometric quantitation of the amount of precipitated CD9 and co-immunoprecipitated integrin β_1_ and ADAM17 (means ± SEM) normalized to the immunoprecipitated CD9 in each of the four independent experiments. Statistical analysis was carried out using two-tailed paired *T*-test. *p < 0.05, **p < 0.01, ***p < 0.001.

To biochemically confirm these data, we immunoprecipitated (IP) α5β1 integrin from Colo320 and Colo320-CD9 cells using mAb Lia1/2, which recognizes an extracellular epitope on the β1 subunit of the integrin, and analyzed by immunobloting the amount of co-immunoprecipitated ADAM17. Importantly, to detect only those interactions of α5β1 and ADAM17 taking place at the plasma membrane, we selectively precipitated only the subset of α5β1 molecules expressed on the surface of cells by incubating intact living cells with the anti-β1 mAb followed by washing the excess unbound antibody prior to cell lysis and IP. As shown in Figure 7A, mature ADAM17 (mADAM17) was significantly more efficiently co-immunoprecipitated with α5β1 from Colo320-CD9 than from Colo320 cells, evidencing that α5β1-ADAM17 association on the cell surface is enhanced by the presence of CD9.

In order to directly probe the presence of CD9, ADAM17 and α5β1 in TEMs, as well as their association, we selectively immunoprecipitated cell surface CD9 (TEM fraction) from intact Colo320-CD9 cells and detected the amount of co-immunoprecipitated α5β1 and ADAM17. Figure 7B shows that when IP of cell surface CD9 was performed in the presence of extracellular Ca^2+^ and Mg^2+^, α5β1 and ADAM17 were efficiently co-immunoprecipitated. Interestingly, induction of conformational changes in α5β1 by the presence of Mn^2+^ resulted in lower amounts of co-immunoprecipitated α5β1 and ADAM17 indicating that, in even in the presence of CD9, some degree of dissociation of these molecules takes place when the integrin conformation is altered.

## Discussion

Integrin α5β1, also named VLA5 or CD49e/CD29, is a major cellular receptor for the extracellular matrix protein fibronectin (Fn) [reviewed in (40)]. As such, this integrin is crucial for cell adhesion to the extracellular matrix and is also critically involved in many cell signaling and migration phenomena, with important implication in tumor invasion and progression (41, 42). Integrin α5β1 binds to its canonical ligand Fn through recognition of the Arg-Gly-Asp (RGD) motif in Fn-type-III module 10 as well as a synergy site in Fn-type-III module 9, contributing to the assembly of Fn molecules into fibrils (4, 43). In addition to binding to its canonical ligand Fn, integrin α5β1 has more recently been reported by different groups to bind specifically to the disintegrin domain of the transmembrane metalloproteinase ADAM17 (5–7, 13). These interactions of α5β1 with ADAM17 can occur between molecules expressed on the same cell (*cis* interactions) or on different cells (*trans* interactions). The α5β1-ADAM17 interactions have been investigated in some detail, both in *in vitro* cell free assays through employment of recombinant proteins (5, 6) and also in cellular assays which collectively confirmed that these interactions support intercellular adhesion, such as the one taking place between tumor and fibroblastic cells (7, 13). Interestingly, in those cell free studies it was observed that the direct interaction between ADAM17 and integrin α5β1 molecules resulted in inhibition of ADAM17 proteolytic activity (6). In cellular experiments with kidney mesangial cells it was established that β1 integrin silencing caused an increase in ADAM17 sheddase activity whereas β1 integrin overexpression resulted in reduced ADAM17 activity (6), confirming the inhibitory role of integrin α5β1 on ADAM17 activity in living cells. Noteworthy, in these studies stimulation of integrin α5β1 with divalent cation Mn^2+^, a potent activator of integrins which induces their extended and open headpiece high-affinity conformation, brought about the dissociation of the α5β1-ADAM17 complex with a concomitant increase in ADAM17 sheddase activity. Thus, it was concluded that it is the inactive form of the integrin α5β1 which becomes selectively engaged in direct interactions with ADAM17, thus keeping low the metalloproteinase activity of this enzyme.

While the functional consequences on ADAM17 shedding activity derived from its association with α5β1 have been investigated, no reports have addressed so far the effects of those interactions on integrin α5β1 adhesive activity. Thus, in the present study we have focused on the functional outcome brought about by treatment with mAb 2A10 (which is directed to the disintegrin domain of human ADAM17) or expression of the tetraspanin CD9, on the adhesive capacity the integrin α5β1. We have demonstrated that treatment with mAb 2A10 or expression of CD9 on the cell surface specifically abrogates the α5β1-mediated adhesion of different types of tumor and leukocytic cells both to its canonical ligand Fn and also to its alternative ligand ADAM17-Fc, a recombinant protein which encompasses all the domains of the extracellular region of human ADAM17 (pro-, catalytic-, disintegrin- and membrane proximal-domain) fused to the Fc constant region of human IgG. Previous reports have shown that α5β1-dependent cell adhesion to its ligand ADAM17 is specifically supported by the disintegrin domain of this recombinant protein (5, 7, 13). The fact that mAb 2A10, specific for the disintegrin domain of ADAM17, not only inhibits the α5β1-mediated adhesion of Colo320 and K562 cells to ADAM17-Fc, but also to Fn, suggests that 2A10 mAb, similarly to CD9 expression, could enhance the *cis* ADAM17-α5β1 interactions on the cell surface.

Integrin adhesive capacity is regulated mainly by two alternative and often complementary mechanisms involving, on the one hand, alterations in the conformation of individual integrin molecules that are reflected by changes in affinity, and on the other hand, modifications in the aggregation and organization of integrin molecules which affect their multivalent avidity for ligands (2, 44, 45). Thus, one possibility to explain the inhibition of α5β1-mediated cell adhesion that is brought about by the expression of CD9 could be that this tetraspanin prevents the acquisition of the extended and open headpiece (i.e., the high affinity) conformation of this integrin. Our results showing that the expression of the HUTS21 epitope is not affected by the presence of CD9 under any of the stimulation conditions tested seem to rule out this possibility. An alternative possibility to account for the observed inhibition of α5β1-mediated cell adhesion is that expression of CD9 produces changes in the organization of integrin molecules on the cell surface that would account for a reduction in integrin avidity. Our confocal microscopy analyses of immunofluorescently-stained integrin α5β1 and ADAM17 show that the pattern of distribution of this integrin is affected both by the expression of CD9 and of ADAM17. Thus, on CD9-negative Colo320 cells integrin α5β1 and ADAM17 molecules are distributed in a high number of small discrete clusters, while upon CD9-expression the localization is somewhat more continuous and the number of clusters per individual cell is reduced. Furthermore, when ADAM17 was knocked-out in Colo320-CD9, α5β1 regained the dotted pattern, correlating with a recovery of the adhesion capacity. However, although interesting, the analyses of the overall distribution of α5β1 and ADAM17 at the plasma membrane by confocal microscopy are limited by the resolution limit of the technique and thus, provide limited insight of the detailed molecular environment of each receptor.

Using a combination of different approaches, including *in situ* proximity ligation assays (PLA), immunofluorescence staining followed by confocal microscopy and biochemical co-immunoprecipitation experiments, we have established that tetraspanin CD9 inhibits α5β1-mediated cell adhesion by reinforcing the *cis* association between ADAM17 and integrin α5β1 on the cell surface. *In situ* PLA have been previously employed by Gooz et al. to confirm and quantitatively assess the strength of the interaction between ADAM17 and integrin α5β1 (6). This technique provides positive signals only when two molecules are in close proximity, typically <40 nm, implying a direct molecular interaction. We have employed PLA to quantitatively analyze the effect of CD9 expression on the association between α5β1 and ADAM17 and our results clearly show that the number of PLA fluorescent dots was significantly higher in CD9-positive than in CD9-negative cells, indicating that the *cis* α5β1-ADAM17 association on the cell surface is enhanced by the presence of CD9. Interestingly, when expression of ADAM17 is knocked-out, CD9 is no longer capable to abrogate integrin α5β1-mediated cell adhesion, strongly pointing out to the formation of trimolecular α5β1:CD9:ADAM17 complexes as the mechanism behind the regulation of both the adhesive activity of α5β1 and the shedding function of ADAM17 by tetraspanin CD9.

Gooz et al. also performed co-immunoprecipitation experiments to confirm the association of integrin α5β1 and ADAM17 (6) and showed that stimulation with extracellular manganese ions (Mn^2+^), a potent inducer of integrin activation (2, 31, 44, 46), decreased the association of these two molecules. We performed co-immunoprecipitation experiments to assess both the influence of CD9 expression and that of integrin activation (under different divalent cations conditions) on the association of α5β1 with ADAM17 molecules selectively on the cell surface. Our immunoprecipitation data clearly show that expression of CD9 greatly enhances the association between α5β1 and ADAM17. Upon integrin activation with Mn^2+^ the amount of α5β1 and ADAM17 that co-immunoprecipitates with CD9 is reduced, but not completely abolished. These biochemical results, together with the PLA data, confirm that expression of CD9 reinforces *cis* interactions of α5β1 and ADAM17 on the cell surface highlighting a dominant role for tetraspanin CD9 in regulating the adhesive activity of integrin α5β1 through a reinforcement of the α5β1-ADAM17 association on the cell surface.

## Author contributions

YM-P and BC performed a large part of experimental work, contributed to the analysis and interpretation of data and made the figures. RR, SL-M, VT, HS and PS-O performed experimental work, analyzed data and discussed results. JG and IL contributed new reagents and aided in interpretation of results. MY-M contributed to design research, analysis of data and interpretation of results. CC planned research, analyzed and interpreted data, discussed results, contributed to the design and final form of figures and wrote the manuscript.

### Conflict of interest statement

The authors declare that the research was conducted in the absence of any commercial or financial relationships that could be construed as a potential conflict of interest.

## Supplementary material

Supplementary Figure 1 The adhesion of HSB2 and Jurkat lymphocytic cell lines to plastic-immobilized ICAM-1-Fc is not affected by the presence of mAb 2A10. Cell adhesion to ICAM-Fc is mediated specifically by the integrin LFA-1 (αLβ2), which is only expressed on some leukocytic cells, including HSB2 and Jurkat cells, but not on K562 or Colo320 cells. In all cases, cells were stimulated with PMA (200 ng/ml) for 2 h, loaded with the fluorescent probe BCECF-AM and then allowed to adhere to plastic-immobilized ligand ICAM1-Fc (20 μg/ml) for 60 min at 37°C in the presence of Mn^2+^ (200 μM). Data show the percentage of adhered cells (means ± SEM of three experiments, performed in triplicates). mAb 2A10 did not exert any statistically significant effect on the LFA-1 mediated cell adhesion to ICAM1-Fc for any of the cell lines as analyzed by two-tailed paired *T*-tests.
