# Supplementary figures and images for: CD9 Controls Integrin α5β1-Mediated Cell Adhesion by Modulating Its Association With the Metalloproteinase ADAM17
Source: Front Immunol. 2018 Nov 5;9:2474. doi: 10.3389/fimmu.2018.02474 (PMC6230984; doi:10.3389/fimmu.2018.02474)

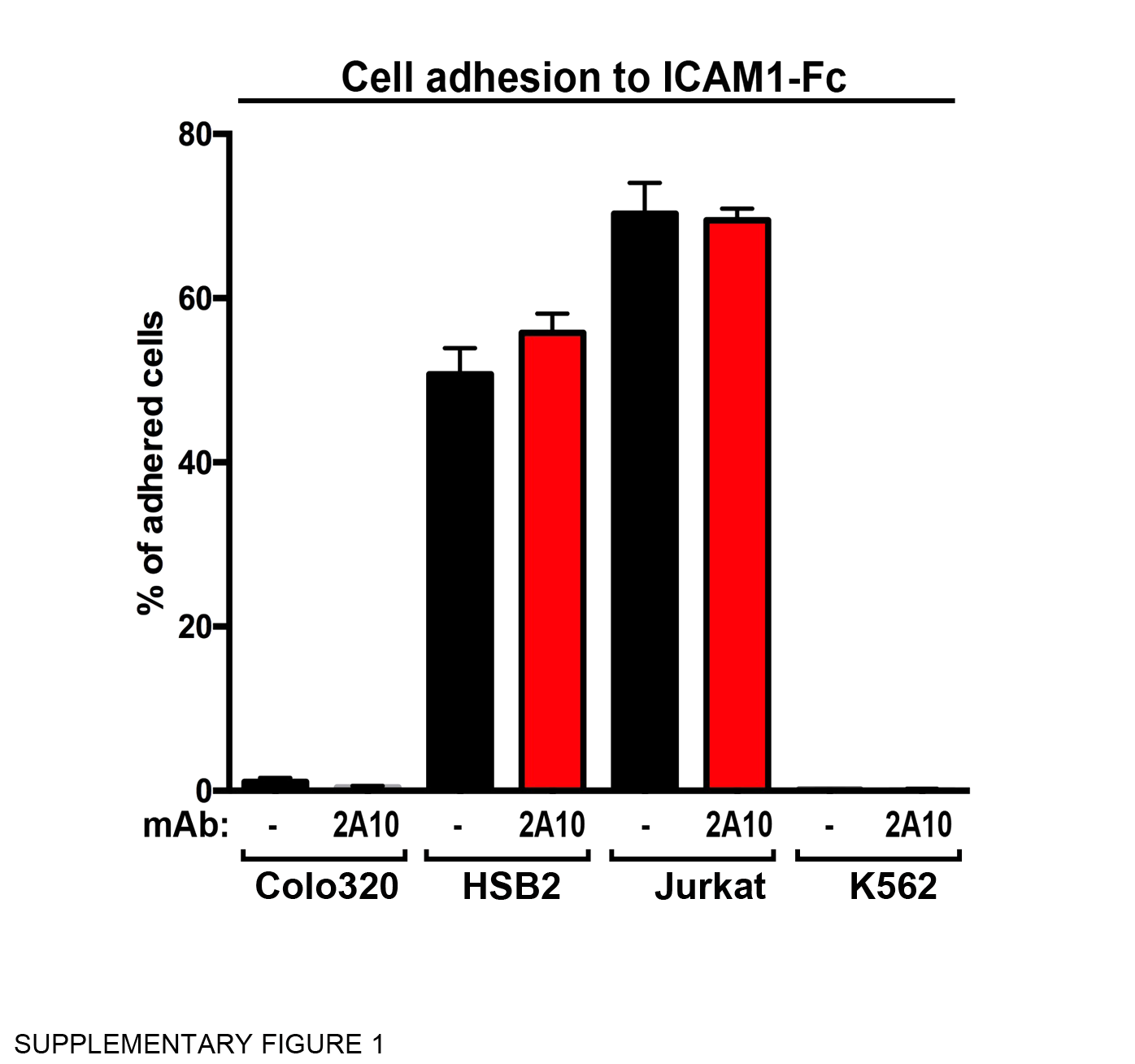

Supplement: Supplementary Figure 1 — The adhesion of HSB2 and Jurkat lymphocytic cell lines to plastic-immobilized ICAM-1-Fc is not affected by the presence of mAb 2A10. Cell adhesion to ICAM-Fc is mediated specifically by the integrin LFA-1 (αLβ2), which is only expressed on some leukocytic cells, including HSB2 and Jurkat cells, but not on K562 or Colo320 cells. In all cases, cells were stimulated with PMA (200 ng/ml) for 2 h, loaded with the fluorescent probe BCECF-AM and then allowed to adhere to plastic-immobilized ligand ICAM1-Fc (20 μg/ml) for 60 min at 37°C in the presence of Mn2+ (200 μM). Data show the percentage of adhered cells (means ± SEM of three experiments, performed in triplicates). mAb 2A10 did not exert any statistically significant effect on the LFA-1 mediated cell adhesion to ICAM1-Fc for any of the cell lines as analyzed by two-tailed paired T-tests. [file Image_1.TIF]
